# Clinical course and outcomes of simultaneous-versus staged-bilateral medial opening wedge high tibial osteotomy

**DOI:** 10.1016/j.asmart.2020.11.003

**Published:** 2020-12-07

**Authors:** Hiroyasu Ogawa, Kazu Matsumoto, Masaya Sengoku, Hiroki Yoshioka, Kyosuke Yamamoto, Tetsuya Shimokawa, Kazuichiro Ohnishi, Haruhiko Akiyama

**Affiliations:** a Department of Orthopaedic Surgery, Ogaki Tokushukai Hospital. Hayashi-machi 6-85-1, Ogaki, 503-0015, Japan; b Department of Orthopaedic Surgery, Gifu University Graduate School of Medicine. Yanagido 1-1, Gifu, 501-1194, Japan

**Keywords:** Simultaneous-bilateral medial opening wedge high tibial osteotomy, Staged-bilateral medial opening high tibial osteotomy, Joint line convergence angle, Alignment, Knee function

## Abstract

**Background:**

Difference in the clinical course and outcomes between simultaneous- and staged-bilateral medial opening wedge high tibial osteotomies (OWHTOs) over time was unknown. The study hypothesis was that patients who underwent simultaneous-bilateral OWHTO (SMBO) have a more rapid improvement in knee function than those who underwent staged-bilateral OWHTO (STBO) due to difference in the change of lower limb alignment between SMBO and STBO.

**Methods:**

The records of 56 knees in 28 patients who underwent either SMBO (n = 28) or STBO (n = 28) were retrospectively analysed. The time course data of weight-bearing line percentage (%WBL), joint line convergence angle (JLCA), and Knee Society Score were compared between the two procedures.

**Results:**

Hospitalisation for SMBO was longer than that for STBO by 1 week. No significant difference was observed in %WBL between the two procedures. The JLCA was significantly lower with SMBO than with the first-stage surgery of STBO (P < 0.05), but it became equivalent in both groups at the last follow-up. The knee scores in both SMBO and the first-stage surgery of STBO significantly improved in approximately 1 year. The function scores in the first-stage surgery of STBO did not significantly improve until the completion of the second-stage surgery whereas those in SMBO significantly improved 1 year after surgery and become stable. The function score 1 year after surgery was significantly higher in SMBO than in the first-stage surgery of STBO (p < 0.001).

**Conclusions:**

Although both SMBO and STBO achieved the desired therapeutic results, SMBO led to earlier functional improvement and decreased JLCA compared with STBO.

## Introduction

Medial opening wedge high tibial osteotomy (OWHTO) is an established surgical option for varus-associated medial compartment osteoarthritis (OA) of the knees. It corrects varus knee alignment and lessens medial compartment loading, resulting in pain relief and knee function recovery. Approximately one out of four patients with OA knee has bilateral disease and requires bilateral surgery.^1^

The traditional lateral closed-wedge high tibial osteotomy has a disadvantage of prolonged rehabilitation with full weight-bearing capacity occurring approximately 6 weeks or more after surgery.^2^ Therefore, staged-bilateral closed-wedge high tibial osteotomy was the only realistic option for patients with bilateral OA knee who required bilateral surgery. Recently, modern OWHTO techniques have led to earlier postoperative full weight-bearing capacity, owing to improved rigid initial fixation by the biplanar osteotomy technique,^3^, ^4^, ^5^ development of a special locking plate,^3^ and reinforcement with bone substitutes.^4^^,^^5^ Takeuchi et al. reported that patients undergoing simultaneous-bilateral OWHTO (SMBO) were able to undergo an early active rehabilitation program and walk with full weight-bearing capacity 3 weeks after surgery.^6^ Hernigou et al. also reported the validity of SMBO on the basis of their analysis of the complications of the surgery.^7^

OWHTO alters lower limb alignment, leg length, and balance, which may affect symptoms and functions of the contralateral knee and, consequently, the ipsilateral knee. Thus, SMBO and staged-bilateral OWHTO (STBO) may influence postoperative clinical course differently. However, the details of the clinical course and therapeutic effects of these two bilateral OWHTOs remain unclear, and understanding them will be useful in selecting either SMBO or STBO for patients with bilateral OA knee. This study retrospectively analysed clinical data from patients who underwent either SMBO or STBO to investigate the procedures’ clinical course and outcomes. The study hypothesis was that SMBO improved knee function among patients earlier than STBO because the first-stage surgery of STBO does not achieve maximal restoration of knee function in patients, which is achieved only after the completion of the second-stage surgery.

## Materials and methods

### Patient selection

This research has been approved by the IRB of the authors’ affiliated institutions. The surgical database of our institution was searched to retrospectively identify all patients who had undergone OWHTO from August 2012 to March 2018. There were 143 patients who consecutively underwent 199 procedures during this period. After excluding patients lost to follow-up, 28 patients (19.6%) who had undergone either SMBO (28 knees) or STBO (28 knees) were included. A matched pair analysis was not considered necessary because no significant difference was found in the mean age and sex ratio between the two groups. All surgeries were performed by either of the two specialist knee surgeons in our institution. The surgical indications for OWHTO were as follows: OA knee with medial compartment involvement or spontaneous osteonecrosis of the knee, diagnosed using radiography and magnetic resonance imaging; impaired performance of activities of daily living due to persistent knee pain after at least 3 months of conservative treatment; and requirement of bony angle correction <15°, as calculated preoperatively. The contraindications were OA knee with involvement of the lateral compartment, symptomatic patellofemoral OA, flexion contracture >15°, anterior or posterior cruciate ligament deficiency, and femorotibial angle >185°. There was no age restriction; all consecutive patients who underwent SMBO or STBO met the established criteria. All patients wished to receive operations in the bilateral sides at the time when the operation was decided. There were no specific indication criteria for SMBT and STBO. The decision to undergo SMBO or STBO was determined based on the patient’s wishes. The first-stage surgery of STBO was performed on the knee with the more severe symptoms, and the timing of the second-stage surgery was decided after discussion with the patients.

Table 1 shows the patients’ characteristics in the two groups. No significant difference was noted between the two procedures with respect to mean age, sex, body mass index, or follow-up period. The change in lower limb alignment and the Knee Society Score (KSS) were compared between the two groups during the course of the clinical study. Each knee was separately evaluated using knee score and function score was for both knees. Since the second-stage surgery of STBO was performed 13.5 ± 7.7 months after the first-stage surgery, the values of parameters at the second-stage surgery of STBO were compared with those recorded at 1 year after SMBO as a reference.

**Table 1:** Patient characteristics.

Variable	SMBO	STBO	P value
Number of patients	14	14	–
Number of knees	28	28	–
Age (years)	62.2 ± 6.2	60.7 ± 6.2	n.s.
Sex (female/male)	9/5	13/1	n.s.
Body mass index (kg/m^2^)	25.4 ± 4.3	26.6 ± 4.2	n.s.
Follow-up period from the first-stage surgery (months)	49.1 ± 22.7	61.8 ± 21.4	n.s.
Follow-up period from the second-stage surgery (months)	–	48.3 ± 20.6	–
Time between the first- and second-stage surgery (months)	–	13.5 ± 7.7	–
Hospitalisation per operation (days)	31.9 ± 7.6	24.3 ± 4.6	<0.001

SMBO, simultaneous-bilateral medial opening wedge high tibial osteotomy; STBO, staged-bilateral medial opening wedge high tibial osteotomy; n.s., not significant.

### Surgical procedure and postoperative rehabilitation

The preoperative planning and surgical procedures were performed as described in previous studies.^8^, ^9^, ^10^ In the preoperative planning, the correction angle was determined by aiming for a postoperative weight-bearing line percentage (%WBL) of 57.5%–62.5% on a long-leg weight-bearing radiograph. Before OWHTO, arthroscopic microfracture for cartilage lesions and meniscal debridement was performed in all patients. The biplanar OWHTO was internally fixed with a TomoFix Osteotomy System (DePuySynthes, Zuchwil, Switzerland) or Tris Medial HTO Plate System (Olympus Terumo Biomaterials, Tokyo, Japan) and two β-tricalcium phosphate wedge spacers (BONISH®, DePuySynthes, Tokyo, Japan, or Osferion60, Olympus Terumo Biomaterials, Tokyo, Japan).^3^^,^^11^^,^^12^ In SMBO, operation was performed the right side first and the left side next.

The postoperative rehabilitation program was the same for both the procedures, and patients were allowed partial and full weight-bearing on postoperative days 7 and 14, respectively. Discharge date was determined based on the prerequisites of safety and stability while walking, with or without a T-cane, and independence in daily activities.

### Clinical evaluation

KSS was used to evaluate clinical outcomes. Patients were followed up at 2 months, 3 months, 6 months, 1 year after surgery, and annually thereafter.

### Radiographic evaluation

Radiographic parameters, including %WBL,^13^ medial proximal tibial angle (MPTA),^12^ posterior tibial slope (PTS),^14^ joint line convergence angle (JLCA),^9^ and Kellgren–Lawrence (K-L) grades,^15^ were evaluated. The leg length was defined as the distance from the centre of the hip joint to the plafond of the ankle on a long-leg weight-bearing radiograph. Leg length discrepancy was shown as a positive value on the right side in SMBO and on the side of the first-stage surgery of STBO.

### Statistical analyses

Two independent observers performed radiographic measurements twice in a blinded manner. Intra- and inter-observer reliability of the measurements were expressed using intraclass correlation coefficients (two-way mixed effects model) that varied from 0 (no agreement at all) to 1 (total agreement). The intra- and inter-observer reliability were greater than 0.88 and 0.85, respectively. Analysis of variance (ANOVA), paired t-test, and Fisher’s exact test were used to analyse inter- and intra-group differences. Statistical analyses were performed using SPSS version 13.0 (IBM, Armonk, NY, USA). P < 0.05 was considered significant.

## Results

### Radiographic evaluation

**Fig. 1:**
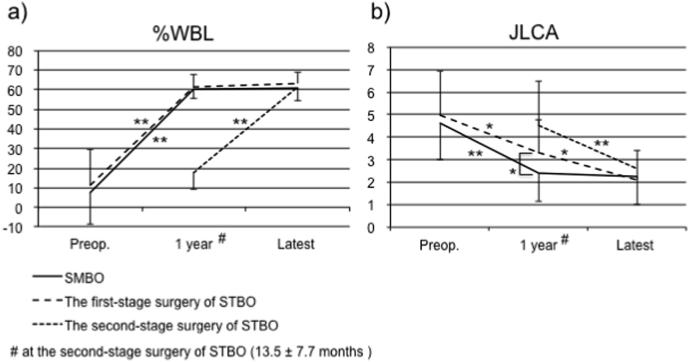
Time course analysis of %WBL and JLCA. a) The %WBL after SMBO and the first-stage and second-stage surgeries of STBO. b) The JLCA after SMBO and the first-stage and second-stage surgeries of STBO. ∗P < 0.05, ∗∗P < 0.01.

No significant differences were noted in pre- and postoperative MPTA and PTS values, leg length discrepancy, or preoperative K-L grades between the SMBO and STBO groups (Table 2, Table 3). %WBL of SMBO and the first-stage surgery of STBO had significantly changed and had become stable 1 year after surgery (Fig. 1a). Although JLCA after SMBO and the first-stage surgery of STBO was significantly lower at 1 year after surgery than preoperatively, JCLA with SMBO was significantly lower than that with the first-stage surgery of STBO at 1 year after surgery (Fig. 1b). However, at the last follow-up, JLCA with both STBO surgeries was comparable to that with SMBO. No patients experienced severe complications requiring re-operation (e.g. deep-seated infection, fracture, deep venous thrombosis, pulmonary embolism, delayed union, or non-union) in either procedure.

**Table 2:** Radiographic evaluation.

Variable	SMBO	STBO	P value
Preoperative MPTA (degree)	81.1 ± 15.2	82.2 ± 14.8	n.s.
Postoperative MPTA (degree)	93.1 ± 2.7	93.8 ± 3.0	n.s.
Preoperative PTS (degree)	6.3 ± 2.7	6.5 ± 2.9	n.s.
Postoperative PTS (degree)	8.0 ± 4.5	7.8 ± 3.6	n.s.
Preoperative K-L grade	3.1 ± 0.5	3.1 ± 0.4	n.s.

SMBO, simultaneous-bilateral medial opening wedge high tibial osteotomy; STBO, staged-bilateral medial opening wedge high tibial osteotomy; MPTA, medial proximal tibial angle; PTS, posterior tibial slope; K-L grade, Kellgren–Lawrence grade; n.s., not significant.

**Table 3:** Leg length discrepancy (mm).

	SMBO	STBO	P-value
Preoperative	2.1 ± 3.6	4.9 ± 4.2	n.s.
Before the second-stage surgery	–	3.8 ± 8.6	–
At the last follow-up	1.0 ± 5.1	0.7 ± 7.7	n.s.

SMBO, simultaneous-bilateral medial opening wedge high tibial osteotomy; STBO, staged-bilateral medial opening wedge high tibial osteotomy; n.s., not significant.

### Clinical evaluation

Hospitalisation per operation was significantly longer for SMBO than for any stage of STBO (p < 0.001), but when both stages of STBO were considered together, the overall hospitalisation was significantly longer for STBO than for SMBO (p < 0.001, Table 1). There were no complications requiring specific treatment such as deep venous thrombosis, deep infection or fractures.

**Fig. 2:**
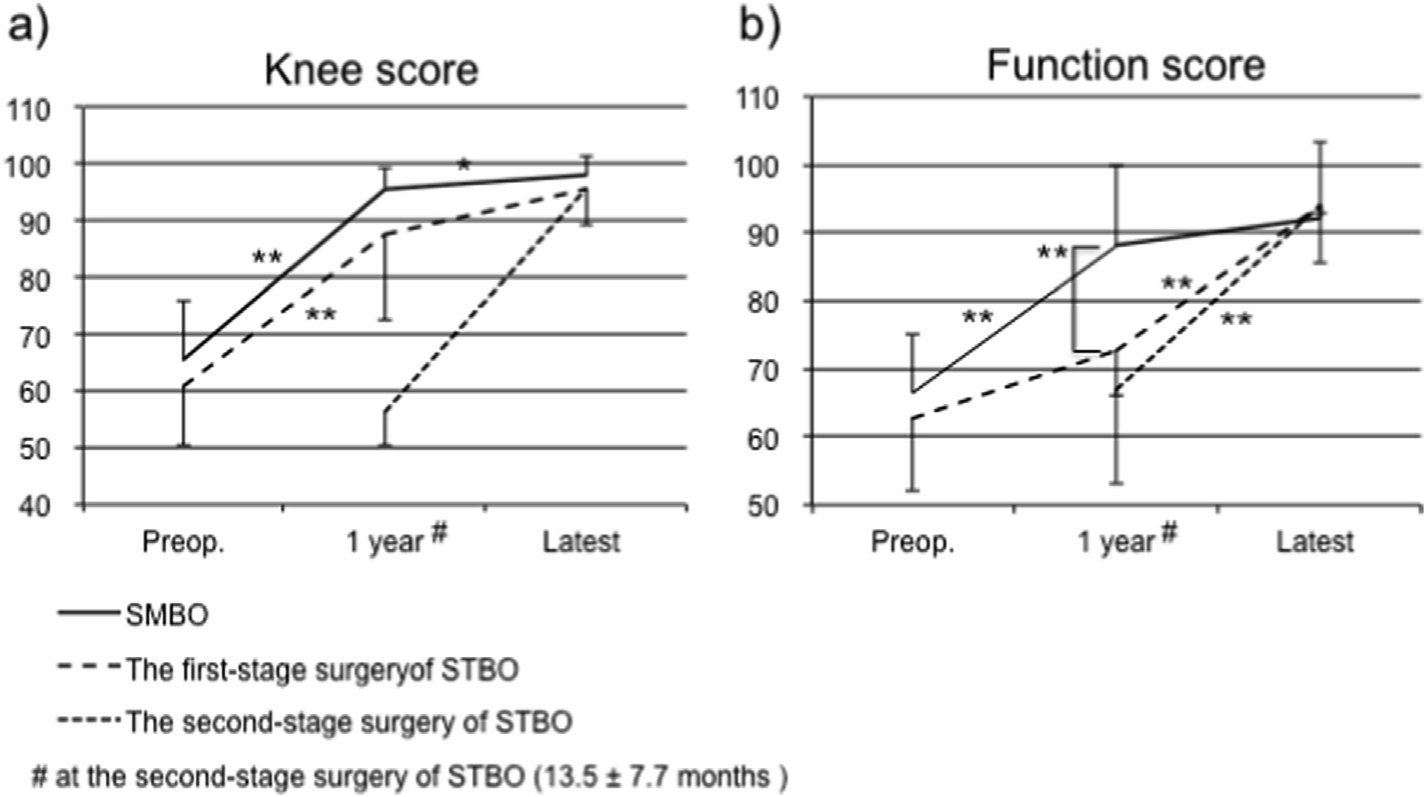
The time course of KSS. a) The knee scores after SMBO and the first-stage and second-stage surgeries of STBO. b) The function score after SMBO and the first-stage and second-stage surgeries of STBO.

The knee scores following SMBO and the first-stage surgery of STBO significantly improved and became stable at approximately 1 year (Fig. 2a). By contrast, the function score after the first-stage surgery of STBO did not show significant improvement until after the second-stage surgery and significantly increased thereafter, whereas that following SMBO significantly improved 1 year after surgery and was stable thereafter (Fig. 2b). The function score of SMBO was significantly higher than that of the first-stage surgery of STBO 1 year after surgery. No significant difference was noted in the function scores between SMBO and both surgeries in STBO at the last follow-up (Fig. 2).

## Discussion

The most important finding of this study was that the function score improved 1 year after SMBO, whereas a significant improvement was not seen with STBO until the second-stage surgery was completed. For patients with bilateral OA knee who wished to undergo bilateral surgery, SMBO may be recommended for a more earlier improvement in knee function with only a 1-week-longer hospitalisation compared with STBO.

Compared with total knee arthroplasty, OWHTO has fewer complications such as deep venous thrombosis^16^ and is indicated for active young patients with OA knee with medial compartment involvement.^17^ Hence, OWHTO is more suitable for simultaneous-bilateral surgery than total knee arthroplasty, but it has a longer postoperative unloading period of the affected limb than total knee arthroplasty, precluding simultaneous surgery. However, in recent years, modern OWHTO techniques have led to early postoperative full weight-bearing capacity,^3^, ^4^, ^5^ and so both knees are operated on at the same time. The advantages of SMBO have been reported previously.^6^^,^^7^ Takeuchi et al. reported that SMBO was superior to STBO in terms of minimising hospitalisation, utilising only a single administration of anaesthesia, reducing costs, and maximising clinical outcomes for patients and institutions.^6^ Similarly, in the current study, hospitalisation with SMBO was only 1 week longer than that with one surgery of STBO, and patients who underwent SMBO achieved full weight-bearing walk and independence in daily activities approximately 4 weeks after surgery. Although there were no severe complications requiring specific treatment, there may be still potential disadvantages of SMBO such as blood loss at one time, operating time at one time, and postoperative pain.^7^ On the basis of our results and previous similar reports,^6^^,^^7^ SMBO is a reasonable option for patients with bilateral OA knee.

There has been little information about the clinical course of SMBO and STBO.^6^^,^^7^^,^^18^^,^^19^ In the current study, the knee and function scores after SMBO significantly improved and became stable 1 year after surgery. However, in STBO, function scores did not improve until after the second-stage surgery was completed in spite of significant improvement in the knee score after the first-stage surgery. There are two possible explanations for this. First, the non-operated knee might have affected total knee function. For example, even if only one knee had improved, it would still have been difficult to climb stairs with a painful knee on the other side. Second, the change in leg length discrepancy due to the first-stage surgery may have affected knee function.^20^ OWHTO increases leg length by approximately 5–7 mm,^21^^,^^22^ and any change in leg length discrepancy adversely affects osteoarthritic knee symptoms in both knees.^20^^,^^23^ In the current study, leg length discrepancy did not change after the first-stage surgery of STBO and became smaller after the second-stage surgery. Meanwhile, SMBO tended to make leg length discrepancy smaller. It is unclear how leg length discrepancy due to STBO actually affects the operated knees, but the change in JLCA seems to be related to knee function. Although JLCA did not significantly change after the first-stage surgery of STBO, it changed significantly after the second-stage surgery, suggesting that appropriate load shift from the medial to the lateral compartment occurred on the first operated knee only after the second-stage surgery. In spite of the change in JLCA, %WBL did not change; this is presumably because the change that occurs in coronal tibiofemoral subluxation due to OWHTO affects the accurate measurement of perioperative %WBL.^12^ These results reveal that the completion of bilateral OWHTO reduced leg length discrepancy and increased function scores in both knees over time. This suggests that the completion of bilateral surgery is necessary for maximal therapeutic effect, including restoration of knee function. Thus, SMBO has faster recovery of knee function than STBO. Surgeons should consider the clinical course and outcomes of each procedure while treating patients with bilateral OA who hope for an earlier recovery of knee function.

This study had some limitations. There may have been unknown selection bias in this retrospective study. Specific surgical indications for bilateral surgery and the timing of the second-stage surgery of STBO were not explicit. Optimal timing of the second-stage surgery of STBO may have altered the time course analysis of KSS in STBO. We analysed the clinical time course data approximately 1 year after surgery or at the last follow-up, so the data at the other time points (e.g. 3 and 6 months after surgery) were unknown. A more detailed clinical time course study is warranted. This retrospective case–control study compared the clinical course of a small number of patients in a short time period. Randomised controlled studies with a larger cohort and longer follow-up periods are necessary to validate our findings. Implants were different among patient because they were selected by surgeon’s preference. The effect of different implants on clinical outcome remains unknown though implants were the similar type of plates.

## Conclusion

Both SMBO and STBO achieved the desired therapeutic results, but compared with STBO, SMBO resulted in rapid functional improvement with only 1 extra week of hospitalisation.

## Declaration of competing interest

The authors have no conflicts of interest relevant to this article.
